# First report of *Aedes albopictus* (diptera: culicidae) for Peru

**DOI:** 10.17843/rpmesp.2026.433.16375

**Published:** 2026-09-01

**Authors:** Carlos Gustavo Tong Rios, Johnny Darío Ramírez Fernández, Clara Rosa Del Águila Morante, Freddy Gutiérrez Rodríguez, José Luis Barboza Chichipe, Becker Amilton Reyna Aspajo, Gladys Giannina Vásquez del Águila, Edwin Requena-Zuñiga

**Affiliations:** 1 Unidad de Entomología del Laboratorio de Referencia Regional, Dirección Regional de Salud, Loreto, Peru. Unidad de Entomología del Laboratorio de Referencia Regional Dirección Regional de Salud Loreto Peru; 2 Dirección de Salud Ambiental, Dirección Regional de Salud, Loreto, Peru. Dirección de Salud Ambiental Dirección Regional de Salud Loreto Peru; 3 Dirección Ejecutiva del Centro de Prevención y Control de Enfermedades Metaxénicas y Zoonosis, Dirección Regional de Salud, Loreto, Peru. Dirección Ejecutiva del Centro de Prevención y Control de Enfermedades Metaxénicas y Zoonosis Dirección Regional de Salud Loreto Peru; 4 Laboratorio de Referencia Nacional de Entomología, Instituto Nacional de Salud, Lima, Peru. Laboratorio de Referencia Nacional de Entomología Instituto Nacional de Salud Lima Peru

Dear Editor. *Aedes* (*Stegomyia*) *albopictus* (Skuse, 1894), commonly known as the “Asian tiger mosquito,” is a species native to Southeast Asia with a high capacity for dispersion and adaptation, which has allowed it to establish globally ^1^. This species is an important vector of more than 20 arboviruses of medical relevance, including dengue ^1^. In the Peruvian context, this vectorial capacity is of particular importance: *Ae. albopictus* is a competent vector of dengue, chikungunya, and Zika viruses —arboviruses with active transmission in the country— and has protagonized large-scale epidemics outside its area of origin, such as chikungunya. Due to its anthropophilic behavior and its simultaneous presence in domestic and wild environments, it is also considered a potential “bridge vector,” capable of facilitating the passage of arboviruses from sylvatic cycles to human populations; an aspect of particular relevance in the Peruvian Amazon, where the sylvatic circulation of the yellow fever virus persists.

Larvae can develop in various natural and artificial breeding sites such as discarded items and those used for water storage ^1^^,^^3^, similarly females exhibit diurnal activity with exophagic and exophilic habits, and feed mainly on birds and mammals, although they also show anthropophilic behavior ^2^^,^^4^. The species has the capacity to transmit dengue serotypes and has been shown to perform transovarial vertical transmission, allowing the emergence of infected individuals without prior contact with humans ^2^. The entry of *Ae. albopictus* into the Americas was in the 1980s, and since then, the species has rapidly expanded throughout several Latin American countries ^3^. Currently, Peru and Chile are the only countries in South America without confirmed records to date ^3^^,^^5^^,^^6^. In Ecuador, the species was first reported in 2017 in the city of Guayaquil ^7^ and, subsequently, in El Coca and Lago Agrio, border localities with Peru ^8^. These areas maintain active commercial exchange through the fluvial route of the Napo River basin, considered as the probable route of introduction of the species into Peru.

For this reason, with the objective of determining the abundance and richness of mosquito species, an entomological monitoring was carried out in localities of the Torres Causana district, Loreto region, located on the border between Peru and Ecuador.

Between September 16 and 24, 2025, house inspections ^9^ of dwellings were carried out in the localities of Cabo Pantoja (-0.9700153, -75.1704073) and Angoteros (-1.5682188, -74.6104670) of the Torres Causana district to search for the larval stages of *Aedes* spp. ^9^^)^ in potential breeding sites. Larvae and pupae were collected and reared until adult emergence. 38 dwellings were inspected in both localities, of which 17 were positive for the species.

Larvae that did not complete their development were sacrificed and preserved in 70% alcohol for their subsequent identification in the Entomology Unit of the Loreto Regional Reference Laboratory, using the entomological keys by Consoli and Oliveira ^10^, and Gyawali ^11^.

The identified specimens showed descriptive characteristics of the genus *Aedes* in their larval and adult stages. Adults of *Ae. albopictus* are black with white ornamentation on the thorax and abdomen; the thoracic scutum presents a well-defined narrow median line of white scales, the clypeus does not present a group of white scales, and the mid-femur without a longitudinal white line. Females show the abdomen ending in a point and smooth claws on the fore and mid tarsi. The mesanepimeron with continuous white scales forming a "V" shape. Larvae showed a comb of spines in a row, which are formed by a single central spine and without subapical spines ^10^^,^^11^ (Figure 1).

**Figure 1:**
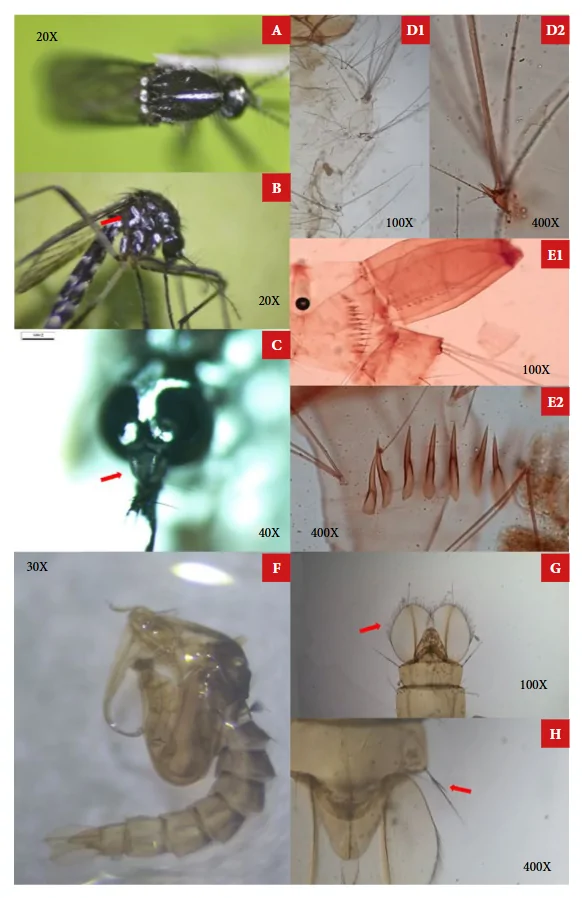
Specimens of *Aedes albopictus* captured in the district of Torres Causana, Loreto region 2025. Adult. A. Thoracic scutum with a well-defined white median line at 20X; B. Mesanepimeron with white scales continuing to form a "V" shape at 20X; C. Clypeus without presence of scales at 32X. Larva. D. Presence of small chitinized spines on both sides of the thorax at 100X and 400X. E. Detail of the comb of the eighth abdominal segment at 100X and 400X. F. Pupal exuvia. G. Paddle visually surrounded by setae. H. Eighth pupal segment with a branched aciculate seta above its base.

The deposits positive for *Aedes* spp. larvae were the barrels used for water storage, abandoned tires, birdbaths, and abandoned and unusable sinks. In all deposits positive for *Ae. albopictus* larvae, the presence of *Aedes aegypti* larvae was observed.

As a result, 555 specimens of *Ae. albopictus* were identified in the locality of Cabo Pantoja: 452 in larval stage and 103 in adult phase obtained from rearing the larval stages. In the locality of Angoteros, 17 specimens were recorded, of which 12 corresponded to larvae and 5 to adults. Likewise, biological material was sent to the Vector Area of the Instituto Nacional de Salud for quality control, confirming that all samples corresponded to *Ae. albopictus*.

The detection of this vector represents an additional challenge for control strategies, as it occupies diverse habitats, including natural environments such as forests, where it can lay its eggs in objects that accumulate water, making its control difficult ^1^^-^^4^. Unlike *Ae. aegypti*, whose distribution is mainly limited by temperature to warm areas, *Ae. albopictus* presents a marked ecological adaptability that allows it to colonize natural, rural, peri-urban, and peri-forest habitats, in addition to urban ones. Its ability to produce desiccation-resistant and cold-tolerant eggs confers upon it the potential to establish itself in higher altitude and more temperate climate areas, such as the inter-Andean valleys, usually less favorable for *Ae. aegypti*, which could significantly expand the arbovirosis risk map in the country. This plasticity, added to its passive dispersion through trade and fluvial and terrestrial transport (for example, in used tires), facilitates its rapid expansion into new areas of the national territory.

Due to the high logistical cost of travel, the inclusion of study sites was strictly limited to larger localities with health centers. This spatial restriction, assumed as a study limitation, also facilitated the collection of a larger volume of samples and provided the necessary infrastructure for the rearing and identification of culicids.

In conclusion, the detection of *Ae. albopictus* in the Loreto region represents an important finding for public health due to its capacity to transmit arboviruses such as dengue. Its establishment could modify the transmission dynamics of these diseases and expand risk areas towards rural and peri-urban zones. Therefore, it is necessary to strengthen entomological surveillance to determine its distribution and expansion in the Loreto region, evaluate its susceptibility to insecticides, and implement integrated control measures. Likewise, studies are required to determine its role in the transmission of dengue and other arboviroses, given that dengue cases have been reported in localities located in the Napo river basin ^(^^12^.

In particular, it is a priority to implement active and sustained entomological surveillance that allows monitoring the dispersion and geographical expansion of the species from the focus of introduction in the Napo River basin towards other localities in the Amazon, as well as towards the coast and the Andean regions. This would allow early detection of its expansion and guide the timely implementation of integrated containment and control measures.
