# Supplementary material for: First report of Aedes albopictus (diptera: culicidae) for Peru
Source: Rev Peru Med Exp Salud Publica. 2026 Sep 1;43(3):415–8. doi: 10.17843/rpmesp.2026.433.16375 (PMC13718570; doi:10.17843/rpmesp.2026.433.16375)
Supplement: Translation

# Translation: Primer reporte de *Aedes albopictus* (diptera: culicidae) para el Perú

**Authors:** Carlos Gustavo Tong Rios, Johnny Darío Ramírez Fernández, Clara Rosa Del Águila Morante, Freddy Gutiérrez Rodríguez, José Luis Barboza Chichipe, Becker Amilton Reyna Aspajo, Gladys Giannina Vásquez del Águila, Edwin Requena-Zuñiga

**Affiliations:** 1 Unidad de Entomología del Laboratorio de Referencia Regional de la Dirección Regional de Salud Loreto, Perú.; 2 Dirección de Salud Ambiental de la Dirección Regional de Salud Loreto, Perú.; 3 Dirección Ejecutiva del Centro de Prevención y Control de Enfermedades Metaxénicas y Zoonosis de la Dirección Regional de Salud Loreto, Perú.; 4 Laboratorio de Referencia Nacional de Entomología. Instituto Nacional de Salud, Lima, Perú

Sr. Editor. *Aedes* (*Stegomyia*) *albopictus* (Skuse, 1894), conocido comúnmente como «mosquito tigre asiático», es una especie originaria del sudeste asiático con alta capacidad de dispersión y adaptación, lo que le ha permitido establecerse globalmente ^1^. Esta especie es un importante vector de más de 20 arbovirus de relevancia médica, incluyendo el dengue ^1^, En el contexto peruano, esta capacidad vectorial reviste especial importancia: *Ae. albopictus* es vector competente de los virus del dengue, chikungunya y Zika -arbovirosis con transmisión activa en el país- y ha protagonizado epidemias de gran magnitud fuera de su área de origen, como la de chikungunya. Por su comportamiento antropofílico y su presencia simultánea en ambientes domésticos y silvestres, se le considera además un potencial «vector puente», capaz de facilitar el paso de arbovirus desde ciclos selváticos hacia las poblaciones humanas; aspecto de particular relevancia en la Amazonía peruana, donde persiste la circulación selvática del virus de la fiebre amarilla.

Las larvas se pueden desarrollar en diversos criaderos naturales y artificiales como inservibles y los utilizados para almacenar agua ^1^^,^^3^, del mismo modo las hembras presentan actividad diurna de hábitos exofágicos y exofílicos, y se alimentan principalmente de aves y mamíferos, aunque también muestran comportamiento antropofílico ^2^^,^^4^. La especie tiene la capacidad de transmitir los serotipos del dengue y se ha comprobado que puede realizar transmisión vertical transovárica, permitiendo la aparición de individuos infectados sin contacto previo con los seres humanos ^2^. El ingreso de *Ae. albopictus* a las Américas fue en la década de los 1980, y desde entonces, la especie se ha expandido rápidamente por varios países de América Latina ^3^. En la actualidad, Perú y Chile son los únicos países en Sudamérica sin registros confirmados hasta la fecha ^3^^,^^5^^,^^6^. En Ecuador, la especie fue reportada por primera vez en 2017 en la ciudad de Guayaquil ^7^ y, posteriormente, en El Coca y Lago Agrio, localidades fronterizas con el Perú ^(^^8^. Estas zonas mantienen un activo intercambio comercial a través de la vía fluvial de la cuenca del río Napo, considerada como la probable ruta de introducción de la especie al Perú.

Por tal motivo, con el objetivo de determinar la abundancia y riqueza de especies de mosquitos, se realizó un monitoreo entomológico en localidades del distrito de Torres Causana, región Loreto, ubicadas en la frontera entre Perú y Ecuador.

Entre el 16 y el 24 de septiembre del 2025, se realizaron inspecciones domiciliarias ^9^ de viviendas en las localidades de Cabo Pantoja (-0.9700153, -75.1704073) y Angoteros (-1.5682188, -74.6104670) del distrito de Torres Causana para la búsqueda de los estados larvarios de *Aedes* spp. ^9^^)^ en potenciales criaderos. Larvas y pupas fueron colectadas y criadas hasta la emergencia de adultos. Se inspeccionaron 38 viviendas en ambas localidades, de las cuales 17 resultaron positivas a la especie.

Las larvas que no completaron su desarrollo fueron sacrificadas y preservadas en alcohol al 70% para su posterior identificación en la Unidad de Entomología del Laboratorio de Referencia Regional de Loreto, utilizando las claves entomológicas de Consoli y Oliveira ^10^, y Gyawali ^11^.

Los ejemplares identificados mostraron las características descriptivas al género *Aedes* en sus estados de larva y adulto. Los adultos de *Ae. albopictus* son de color negro con ornamentaciones blancas en el tórax y abdomen; el escudo torácico presenta una línea estrecha media bien definida de escamas blancas, el clípeo no presenta grupo de escamas blancas y el fémur medio sin una línea de longitudinal blanca. Las hembras muestran la terminación del abdomen en punta y uñas lisas en las tarsos anteriores y medios. El mesanepímero con escamas blancas continúas dando una forma de «V». Las larvas mostraron tener un peine de espinas en hilera, las mimas que están formadas por una sola espina central y sin espinas subapicales ^10^^,^^11^ (figura 1).

**Figura 1:** Ejemplares de *Aedes albopictus* capturados en el distrito de Torres Causana, región de Loreto 2025. Adulto. A. Escudo torácico con una línea media blanca bien definida a 20X; B. Mesanepímero con escamas blancas continúa dando una forma en «V» a 20X; C. Clípeo sin presencia de escamas a 32X. Larva. D. Presencia de pequeñas espinas quitinizadas en ambos lados del tórax a 100X y 400X. E. Detalle del peine del octavo segmento abdominal a 100X y 400X. F. Exsuvia de pupa. G. Paleta visualmente rodeadas de cerdas. H. Octavo segmento de la pupa con una cerda aciculada ramificada por encima de su base.

Los depósitos positivos a larvas de *Aedes* spp. fueron los bidones utilizados en el almacenamiento de agua, neumáticos abandonados, bebederos para aves, lavatorios abandonados e inservibles. En todos los depósitos positivos para larvas de *Ae. albopictus* se observó la presencia de larvas de *Aedes aegypti*.

Como resultado, en la localidad de Cabo Pantoja se identificaron 555 ejemplares de *Ae. albopictus*: 452 en estadio de larva y 103 en fase adulta obtenidas de la crianza de los estados larvarios. En la localidad de Angoteros se registraron 17 ejemplares, de los cuales 12 correspondieron a larvas y 5 a adultos. Asimismo, se remitió material biológico al Área de Vectores del Instituto Nacional de Salud para su control de calidad, confirmándose que todas las muestras correspondían a *Ae. albopictus*.

La detección de este vector representa un desafío adicional para las estrategias de control, ya que ocupa diversos hábitats, incluyendo ambientes naturales como el bosque, donde puede colocar sus huevos en objetos que acumulen agua, dificultando su control ^1^^-^^4^. A diferencia de *Ae. aegypti*, cuya distribución está limitada principalmente por la temperatura a zonas cálidas, *Ae. albopictus* presenta una marcada adaptabilidad ecológica que le permite colonizar hábitats naturales, rurales, periurbanos y periforestales, además de los urbanos. Su capacidad para producir huevos resistentes a la desecación y tolerantes al frío le confiere el potencial de establecerse en zonas de mayor altitud y clima más templado, como los valles interandinos, habitualmente menos favorables para *Ae. aegypti*, lo que podría ampliar de manera significativa el mapa de riesgo de arbovirosis en el país. Esta plasticidad, sumada a su dispersión pasiva a través del comercio y del transporte fluvial y terrestre (por ejemplo, en neumáticos usados), facilita su rápida expansión hacia nuevas áreas del territorio nacional**.**

Debido al alto costo logístico de desplazamiento, la inclusión de sitios de estudio se limitó estrictamente a localidades de mayor tamaño con centros de salud. Esta restricción espacial, asumida como limitación del estudio, facilitó a su vez la recolección de un mayor volumen de muestras y proporcionó la infraestructura necesaria para la crianza e identificación de culícidos.

En conclusión, la detección de *Ae. albopictus* en la región Loreto presenta un hallazgo de importancia para la salud pública debido a su capacidad para transmitir arbovirus como dengue. Su establecimiento podría modificar la dinámica de transmisión de estas enfermedades y ampliar las áreas de riesgo hacia zonas rurales y periurbanas. Por ello, es necesario fortalecer la vigilancia entomológica para determinar su distribución y expansión en la región Loreto, evaluar su susceptibilidad a los insecticidas e implementar medidas integradas de control. Asimismo, se requieren estudios que permitan determinar su papel en la transmisión del dengue y otras arbovirosis, dado que se han reportado casos de dengue en localidades ubicadas en la cuenca del río Napo ^(^^12^.

En particular, resulta prioritario implementar una vigilancia entomológica activa y sostenida que permita monitorear la dispersión y expansión geográfica de la especie desde el foco de introducción en la cuenca del río Napo hacia otras localidades de la Amazonía, así como hacia la costa y las regiones andinas. Esto permitiría detectar tempranamente su expansión y orientar la implementación oportuna de medidas de contención y control integrado.
